# Analyzing Structural Optical and Phonon Characteristics of Plasma-Assisted Molecular-Beam Epitaxy-Grown InN/Al_2_O_3_ Epifilms

**DOI:** 10.3390/nano15040291

**Published:** 2025-02-14

**Authors:** Devki N. Talwar, Li Chyong Chen, Kuei Hsien Chen, Zhe Chuan Feng

**Affiliations:** 1 Department of Physics, University of North Florida, 1 UNF Drive, Jacksonville, FL 32224, USA; 2 Department of Physics, Indiana University of Pennsylvania, 975 Oakland Avenue, 56 Weyandt Hall, Indiana, PA 15705, USA; 3 Center for Condensed Matter Sciences, National Taiwan University, Taipei 10617, Taiwan; chenlc@ntu.edu.tw; 4 Institute of Atomic and Molecular Sciences, Academia Sinica, Taipei 10617, Taiwan; chenkh@pub.iams.sinica.edu.tw; 5 Southern Polytechnic College of Engineering and Engineering Technology, Kennesaw State University, Marietta, GA 30060, USA; zfeng6@kennesaw.edu

**Keywords:** PA-MBE growth, InN/sapphire epifilms, photoluminescence, Fourier transform infrared, Raman scattering spectroscopy, Drude–Lorentz three-phase model

## Abstract

The narrow bandgap InN material, with exceptional physical properties, has recently gained considerable attention, encouraging many scientists/engineers to design infrared photodetectors, light-emitting diodes, laser diodes, solar cells, and high-power electronic devices. The InN/Sapphire samples of different film thicknesses that we have used in our methodical experimental and theoretical studies are grown by plasma-assisted molecular-beam epitaxy. Hall effect measurements on these samples have revealed high-electron-charge carrier concentration, η. The preparation of InN epifilms is quite sensitive to the growth temperature T, plasma power, N/In ratio, and pressure, P. Due to the reduced distance between N atoms at a higher P, one expects the N-flow kinetics, diffusion, surface components, and scattering rates to change in the growth chamber which might impact the quality of InN films. We believe that the ionized N, rather than molecular, or neutral species are responsible for controlling the growth of InN/Sapphire epifilms. Temperature- and power-dependent photoluminescence measurements are performed, validating the bandgap variation (~0.60–0.80 eV) of all the samples. High-resolution X-ray diffraction studies have indicated that the increase in growth temperature caused the perceived narrow peaks in the X-ray-rocking curves, leading to better-quality films with well-ordered crystalline structures. Careful simulations of the infrared reflectivity spectra provided values of η and mobility μ, in good accordance with the Hall measurements. Our first-order Raman scattering spectroscopy study has not only identified the accurate phonon values of InN samples but also revealed the low-frequency longitudinal optical phonon plasmon-coupled mode in excellent agreement with theoretical calculations.

## 1. Introduction

Narrow bandgap indium nitride (InN) of $E_{g}(\sim 0.7\;eV)$, with smaller effective mass $m_{e}^{*}$ (≡0.04 $m_{e}$–0.14 $m_{e}$), high electron mobility μ (~4400 ${cm}^{2}\;V^{-1}\;s^{-1}$), and large electron drift velocity ($\sim 4.2\times 10^{7}\;cm/s$), represents one of the most important classes of direct bandgap III-N [BN (6.5 eV), AlN (6.2 eV), GaN (3.4 eV)] materials [1,2,3,4,5,6,7]. Due to high chemical, mechanical, and thermal stability, conventional GaN and AlN have been intensively studied over the past four decades [8,9,10,11,12,13,14,15], while InN was the least investigated [16,17,18,19,20] until recently. Most of these compound semiconductors occur either in the hexagonal wurtzite (wz) structure, referred to as an α-type, or in the cubic zinc-blende (zb) structure signified as a β-type [1,2,3,4,5,6,7]. The more thermodynamically stable crystals of the α-phase (B_4_) are expressed either by using a space group $P6_{3}mc$ in the Hermann–Mauguin representation [1,2,3,4] or with $C_{6v}^{4}$ in the Schoenflies notation [5,6]. The β-type (B_3_) zinc-blende (zb) crystal structures with space group $F\bar{4}3m$ have been stabilized if epitaxially grown on the GaAs and/or Si substrates.

Earlier, theoretical predictions [12,13,14], followed by experimental verifications [15,16,17,18,19,20,21], have provided strong evidence for the wz GaN and AlN materials being pyroelectric. Saturated drift velocity, spontaneous electric field-, and polarization-induced high-electron sheet charges have allowed the integration of III-Ns into different devices capable of high-power, high-voltage, and high-temperature operations [12]. Recent developments of improvements in the epitaxial growth of III-Ns [22,23,24,25,26,27,28,29,30,31,32,33,34] have led to the production of light-emitting diodes (LEDs); ultraviolet (UV) lasers; infrared (IR)/UV photodetectors; night vision systems; thermal imaging cameras; mid-infrared lasers; high-electron-mobility transistors (HEMTs); laser diodes (LDs); and solar cells. These devices are being integrated into nanoelectronics/photonics to achieve the next generation of IR-based optical communication networks for operations in λ = 1.55 μm.

Despite the many positive attributes of traditional III-Ns, the design of various device structures based on InN- and/or In-rich alloys has not yet been fully exploited. It is due to their low dissociation temperature and lack of suitable substrates in terms of both the lattice constant and thermal expansion coefficient which resulted in ambiguous values of several experimentally obtained material parameters [12,22,23,24,25,26,27,28,29,30,31,32,33,34]. The literature shows a wide scattering of data [1,2,3,4,5,6,7] on the different fundamental properties of InN including $E_{g}$, $m_{e}^{*}$, mobility μ, and charge carrier concentration η. Conclusive reasoning of the high η values ~10^18^–10^20^ cm^−3^ has not yet been fully ascertained. Growing In-rich ${In}_{x}{Ga}_{1-x}N$ and the formation of phase separation are additional challenges [13,14,15]. At high growth pressures, the N dissociation from the epitaxial layer can be suppressed [13,14,15,16,17,18,19]. These and many other issues of InN and ${In}_{x}{Ga}_{1-x}N$ alloys, linked with their structural, optical, phonon, and electrical properties, are still poorly understood and need to be addressed.

Improvements over the last two decades in the growth and characterization methods have helped us comprehend the fundamental properties of III-Ns to design different device structures for high-speed electronics and photovoltaic applications. Recent reports have indicated that InN films meet the requirements of perceiving practical devices for various strategic and civilian application needs [1,2,3,4,5,6,7]. Using different III-N materials, many review papers, monographs, and books are available which cover the basic characteristics and fabrication of low-dimensional heterostructure (LDH)-based devices [12,13,14,15,16]. Research groups around the world have employed different growth techniques to prepare III-N epifilms on sapphire (Al_2_O_3_), Si, and/or GaAs substrates. The most common methods that are used for the growth include metal–organic vapor phase epitaxy (MOVPE) [22,23,24,25,26], molecular-beam epitaxy (MBE) [27,28,29,30,31,32] sputtering [33,34], hydride vapor phase epitaxy (HVPE) [35,36], liquid phase epitaxy (LPE) [34], reactive magnetron sputtering [33,34], and pulsed laser deposition (PLD) methods [33]. The MBE approach can be broken down into ammonia-based MBE (NH_3_-MBE) and plasma-assisted MBE (PA-MBE). The ammonia-based MBE is often favored over PA-MBE as the growth rate is much higher and closer to that of the MOVPE [22,23,24,25,26] method. Higher growth rates tend to be favored as they reduce the number of impurities incorporated into epilayers, thus having less defects within the device structures. Due to the many advantages of MBE [27,28,29,30,31,32], including its versatility, precise control of beam fluxes, growth conditions, and clean environment, many crystal growers have preferred using it to prepare InN- and InGaN-based LDHs for exploring their basic traits [37,38,39,40,41,42,43,44]. The InN/Al_2_O_3_ epilayers that we have used in our experimental measurements to assess the structural, phonon, and electronic properties were grown by using the SVTA (SVT-V-2) PA-MBE system.

Different characterization methods have been employed to understand the fundamental properties of III-N epilayers. The techniques that are frequently used include the scanning transmission electron microscopy with cathodoluminescence (STEM-CL) [45], high-resolution X-ray diffraction (HR-XRD) [46,47,48,49,50,51,52,53,54,55,56], scanning electron microscopy (SEM) [27,28,29,30,31,32], UV-Vis spectrophotometry [56], near-field scanning optical microscopy [27,28,29,30,31,32], infrared (IR), Raman scattering spectroscopy, photoluminescence (PL), and spectroscopic ellipsometry (SE) [57,58,59,60,61,62,63,64,65,66,67]. From the complementary PL, Raman scattering, and IR measurements on InN/Sapphire epifilms, one expects to acquire a better understanding of their structural, electronic, and vibrational traits. At the same time these attributes are considered a criterion to assess charge carrier concentration, crystalline quality, and surface morphology. Studies on PA-MBE-grown InN films have ascertained narrow bandgaps, rendering the PL emission between 0.6 eV and 0.8 eV in sharp contrast to the higher E_g_ values cited before [44]. Although not intentionally doped, the electron charge carrier density η in as-grown samples is higher (~10^18^–10^20^ cm^−3^) [50,51]. Very little attention has been paid to the nitridation process in contrast to the numerous efforts made to optimize the growth conditions for low T buffer and/or main InN layers. While the narrow bandgap InN epilayers have insinuated better crystalline quality, the origin of large η and its effect on the electronic/vibrational properties has not been fully understood. Moreover, the influence of growth pressure P to prepare InN epifilms has not been studied in detail yet, but it is believed to be a critical parameter [27,28]. At a higher P, due to a reduced distance between N atoms, one expects their flow kinetics, diffusion, surface components, and scattering rate in the growth chamber to change, which might impact the quality of InN films.

This paper aims to report the results of systematic experimental (cf. Section 2, Section 3 and Section 4) and theoretical (cf. Section 5) studies on InN/sapphire epifilms to comprehend their structural, electrical, optical, and phonon properties. The samples used in different experimental (viz., HR-XRD, PL, Optical Absorption, Raman scattering, and infrared) measurements were grown (Section 2.1, Section 2.2 and Section 2.3) by using the SVTA (SVT-V-2) PA-MBE system. Hall experiments were performed to determine the electrical properties of all samples (cf. Section 3 and Section 4), including the charge carrier concentration η and mobility μ. Our results clearly indicated that the InN epilayers are n-type with carrier concentrations η and mobilities μ in the range of 10^18^ cm^−3^ to 10^20^ cm^−3^ and ~ 100 cm^2^/Vs to 987 cm^2^/Vs, respectively. In Section 3.1, Section 3.2 and Section 3.3, the influence of RF N-plasma on the nitridation process is studied to comprehend the growth process of InN films. To measure the band gap $E_{g}$ of InN/Sapphire epilayers, a thermal Fisher IR spectrometer was employed to profile the PL intensities by using an excitation source of 532 nm (cf. Section 3 and Section 4). A thermal micro-Raman scattering spectroscopy system was used with a 633 nm laser to assess the phonon characteristics of InN/Sapphire epifilms (cf. Section 4.2, Section 4.3 and Section 4.4). Besides the PL and Raman spectroscopic studies, the room temperature Fourier transform infrared (FTIR) reflectivity measurements were achieved by using a high-resolution Bruker IFS 120 v/S (cf. Section 4.4) spectrometer. The experimental results are carefully analyzed theoretically (cf. Section 5) by using a “three-phase model” [68,69,70,71,72,73,74] to comprehend the structural, optical, and phonon characteristics of the PA-MBE-grown InN/Sapphire samples. A comparison made between the experimental results and theoretical data is reported in Section 5 for the phonon characteristics, and the optical results on binary InN epifilms have provided a very good agreement. Summaries and conclusions are drawn in Section 6.

## 2. Structural Properties

### 2.1. Crystal Structure

**Figure 1:**
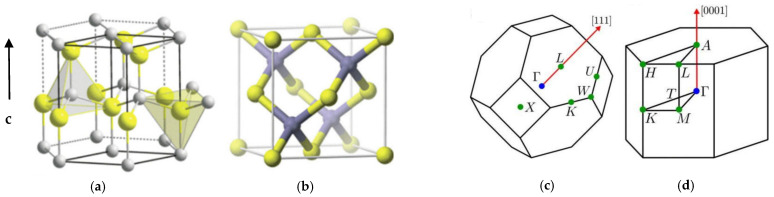
Schematic representation of the two crystal phases of InN and its Brillouin zones. The crystal structures of the (**a**) wurtzite wz, and (**b**) zinc-blende zb InN are shown on the left-hand side, where the small, gray-colored circles represent N atoms while the bigger, yellow-colored circles indicate In atoms. The Brillouin zones of (**c**) zb and (**d**) wz InN are displayed on the right-hand side.

III-N semiconductor materials can occur in the hexagonal wz and/or cubic zb structures (see Figure 1a,b). The unit cell of wurtzite structures consists of hexagonal sublattices, each containing six atoms of the respective elements. The structure of wz materials (see Figure 1a) is non-centrosymmetric because it confers piezoelectric and pyroelectric character. The arrangement of this structure gives polar symmetry along the c-axis, a key factor to crystal growth, etching and defect generation. An epitaxially grown zb structure has been achieved by using certain cubic (Si, GaAs, etc.) substrates [12]. The Brillouin zone (BZ) of a wz structure (Figure 1d) is hexagonal, while the BZ of a zb structure is cubic (Figure 1c), with a key difference being the symmetry of the crystal.

### 2.2. PA-MBE Growth of InN/Sapphire

Epitaxial growth of InN films was achieved on c-plane sapphire substrates by the SVTA (SVT-V-2) PA-MBE system using an RF plasma source for N and the conventional Knudsen effusion cell for indium, In. The sample run numbers of a selected set of InN/Sapphire epifilms are given in Table 1.

During the growth of 48 samples, the N_2_ flow rate was set to stabilize nitrogen’s partial pressure (P) in the growth chamber between 3 × 10^−5^ and 7 × 10^−5^ Torr. After the nitridation process, the N_2_ plasma-power level varied on sapphire substrates from 200 W to 400 W. An ion-trapper was used to deflect the high-energy ion species that induce defects in the epitaxial layers [30]. The InN/Sapphire growth was proceeded by different steps. First, the substrate temperature (T) was increased to 800 °C for 30 min, and the temperature of In Knudsen effusion cell was increased to 850 °C to clean the impurities on the substrate surface in the growth chamber. Second, the nitridation process was applied for surface pretreatment and modification, at 200 °C and under 5.0 × 10^−5^ Torr with N_2_-plasma power set at 400 W for 30 min, which also helped to form a thin AlN buffer layer a few nm thick. Finally, a series of InN thin epifilms was grown using different plasma powers, and a growth P of 3 × 10^−5^ to 7 × 10^−5^ Torr with substrate T of 320 °C and indium flux of 0.9 to 1.2 sccm, maintaining the T between 825 and 846 °C, for 60 min. During the preparation of InN/Sapphire samples, the reflection high-electron energy diffraction (RHEED) was used for monitoring each step to ensure a smooth surface and perfect epitaxial growth. Different characterizations including the Rutherford backscattering [31], X-ray photoelectron spectroscopy, a synchrotron radiation X-ray absorption near-edge structure, SE, and SEM [32] were used to comprehend the structural and electronic properties.

### 2.3. Scanning Electron Microscopy

**Figure 2:**
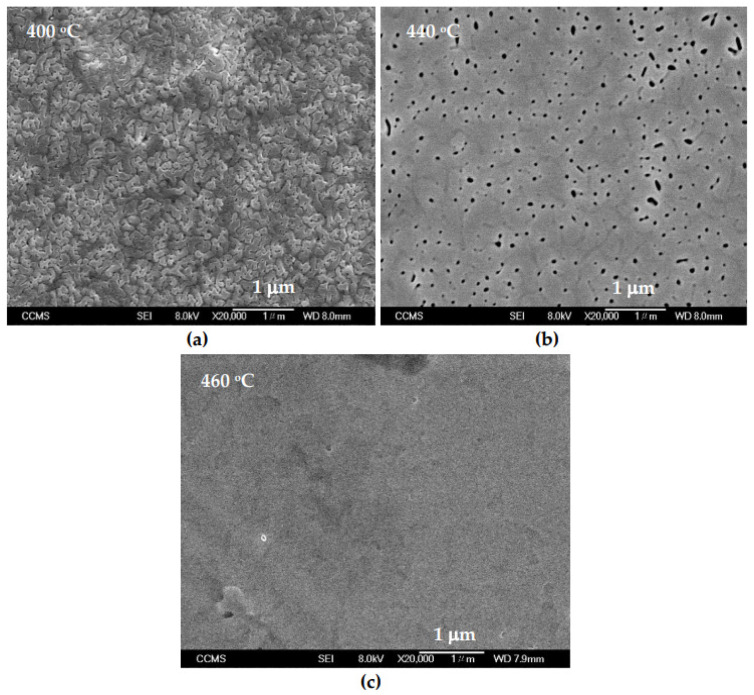
The scanning electron microscopic images recorded at different growth temperatures on one of our PA-MBE-grown samples CC28 at (**a**) 400 °C growth temperature, (**b**) 440 °C growth temperature, and (**c**) 460 °C growth temperature.

The surface morphology of InN/Al_2_O_3_ films was examined by using field-emission scanning electron microscopy (FESEM, JEOL-6700, JEOL Ltd., Tokyo, Japan) [32]. In Figure 2a–c, we have displayed our SEM results on one of the PA-MBE-grown samples CC28. As the growth temperature of InN epitaxial film increases from 400 °C to 460 °C, the surface morphology improves, changing from a rough textured to a smoother epifilm. 

## 3. High Charge Carrier Concentration in InN

Despite attaining the best growth temperature for preparing PA-MBE InN/Sapphire epifilms of different film thicknesses, our Hall measurements (see Table 1) on these samples have revealed a high electron charge carrier concentration, η. The RT data on η were acquired in the van der Pauw configuration using a magnetic field of 0.32 Tesla. One must note that the crystal quality of epitaxial layers and other traits including the polarity are quite sensitive to the nitridation process. Relatively little attention has been paid in contrast to the numerous efforts made to optimize the growth condition for low T buffer/main layers. Here, we have systematically studied (cf. Section 3.2) the impact of N-plasma power on InN growth at T above its decomposition temperature.

### 3.1. Temperature-Dependent van der Pauw Measurements

**Figure 3:**
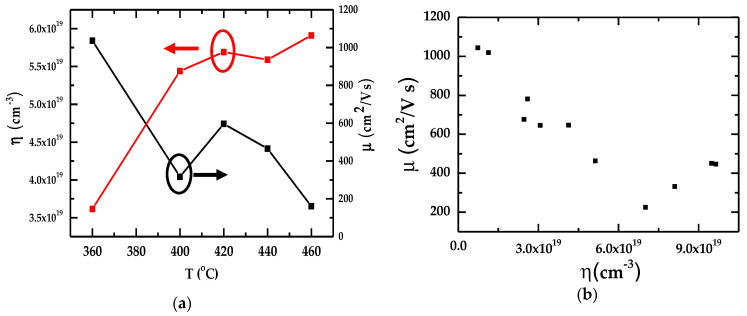
(**a**) Relationship of carrier concentration, η (red squares) and mobility μ (black squares), with the increase in growth temperature T (°C) of an InN/Sapphire epifilm; (**b**) Correlation between the measured electron mobility μ and the carrier concentration, η.

In Figure 3a we have reported our results of Hall measurements on η, μ by increasing the growth T, while in Figure 3b, the relationship between μ and η is displayed.

Obviously, as T increases, the η values increase and μ tends to decrease due to the increased lattice vibrations at higher temperatures, causing more scattering of charge carriers and thereby reducing μ. In exploring the cause of unintentional background doping, we have made experimental efforts (see Section 3.2 and Section 3.3) to comprehend this important problem and provide possible explanations.

### 3.2. Impact of N-Plasma Power on Optical Emission Spectra

**Figure 4:**
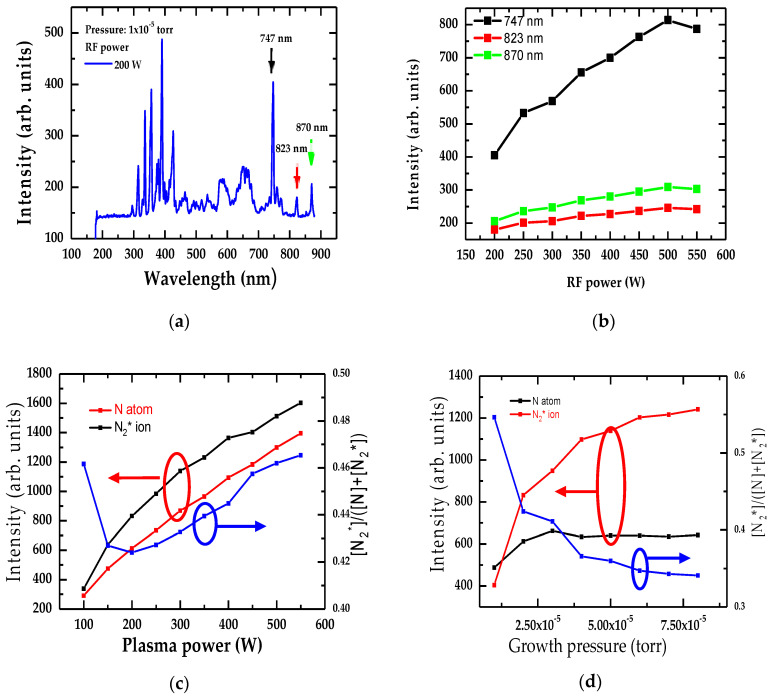
(**a**) Typical optical emission spectra of N plasma with 1 × 10^−5^ torr pressure and RF power 200 W. (**b**) Optical emission spectra for the strongest 747 nm, 823 nm, and 870 nm N lines as a function of the RF power between 200 W and 550 W and 1 × 10^−5^ torr pressure. (**c**) The impact of plasma power on the optical emission spectral intensity of different N-species: the red line (black line) indicates the N atom ($N_{2}^{*}$ ion) while the blue line represents the $N_{2}^{*}$/(N+ N_2_) ratio. (**d**) Same key as (**c**) but for the optical emission spectral intensity as a function of the growth pressure.

In GaN, AlN, and InN, the impact of RF plasma power on N species has been reported in various studies using quadrupole mass spectrometry [34] and optical emission spectroscopy [35,36,37]. The typical optical emission spectrum of N RF-plasma has revealed atomic and molecular N species. In the PA-MBE growth of InN [28,29], it has been advocated that higher RF power generated accelerated N atoms and $N_{2}^{*}$ ions which could severely impact structural and optical traits. By systematically studying the optical emission spectra, we have examined the links between RF power, growth P (see Figure 4a–d) on the intensity of N-atoms, $N_{2}^{*}$ ions, and their ratio of $N_{2}^{*}/(N+N_{2}^{*})$ in the growth of InN.

In Figure 4a, the typical wavelength-dependent results of the optical emission spectra are displayed for an InN/Sapphire sample prepared at RF-plasma power 200 W and pressure 1 × 10^−5^ torr. Focus is placed on the strong atomistic peaks located at 747 nm, 823 nm, and 870 nm due to the strong correlation between the InN film growth rate and N atomic species, as well as $N_{2}^{*}$-ion fluxes [34,35,36].

The variation in plasma power (see Figure 4b) has revealed an increase in intensity for the 747 nm line, while the lines located at 823 nm and 870 nm become saturated for power above 500 W. In Figure 4c, we have noticed a decrease in intensity of the N-atom and $N_{2}^{*}$-ion with a decrease in plasma power, while the ratio $N_{2}^{*}/(N+N_{2}^{*})$ increases with an increase in plasma power, suggesting that it might not be advisable to grow InN epifilms at a higher plasma power. At power < 100 W, the ratio $N_{2}^{*}/(N+N_{2}^{*})$ dramatically increases. In Figure 4d, we have also explored the intensity spectra of $N,\;N_{2}^{*},$ and $N_{2}^{*}/(N+N_{2}^{*})$ by changing the N-pressure. The results showed that the intensity of N-atom increases with an increase in growth pressure while that of $N_{2}^{*}$-ion increases first with similar trends, then it becomes saturated at pressure > 3 × 10^−5^ torr. Consequently, the $N_{2}^{*}/(N+N_{2}^{*})$ ratio decreases dramatically between 1 × 10^−5^–4 × 10^−5^ torr and continues to further decrease gradually.

### 3.3. Impact of N-Plasma Power on Charge Carrier Concentration

**Figure 5:**
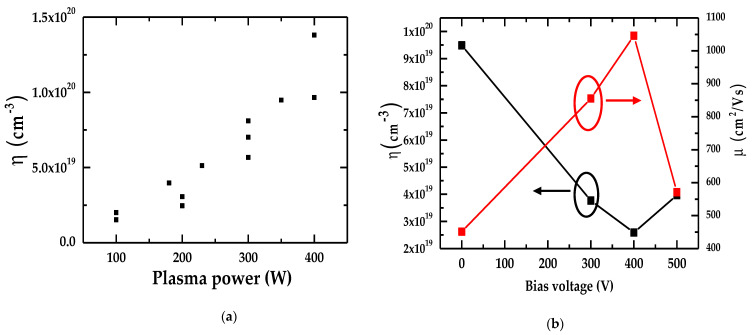
(**a**) The correlation between the electron charge carrier concentration η by increasing the plasma power from 100 W to 400 W, and (**b**) the association between the charge carrier concentration, η, and mobility, μ, with the bias voltage (V).

To assess the relationship between electron charge carrier concentration η and plasma power in InN/Sapphire epifilms, we have displayed, in Figure 5a, the Hall measurements achieved by increasing plasma power from 100 W to 400 W. In Figure 5b, the impact on η and μ by changing the bias voltage between 100 V and 500 V is also reported.

The results of Figure 5a clearly show a decrease in η that was achieved by decreasing the plasma power from 400 W to 100 W. This trend is well corroborated with our earlier results on optical spectra of N plasma power. It implies that $N_{2}^{*}$ ions play important roles inside the PA-MBE chamber during the growth of InN/Sapphire thin films. From Figure 5b, one can also notice that carrier concentration η decreases (by an order of magnitude) from 1 × 10^20^ cm^−3^ to 2 × 10^19^ cm^−3^ by tuning the bias voltage from 0 V to 400 V. This result has given powerful evidence identifying the impact of N-ion on η during the PA-MBE growth of InN/Sapphire epifilms. However, there was an increase in the carrier concentration when the voltage was above 400 V. This may be due to the difference in chamber size causing different reflections of the ions.

## 4. Experimental Characterization

High-resolution X-ray diffraction measurements were carried out in the theta-two-theta coupled geometry by using Cu Kα_1_ radiation. The configuration of reciprocal space mapping in HR-XRD studies was used to determine the lattice constants of the wz InN materials.

### 4.1. HR-XRD Measurements

**Figure 6:**
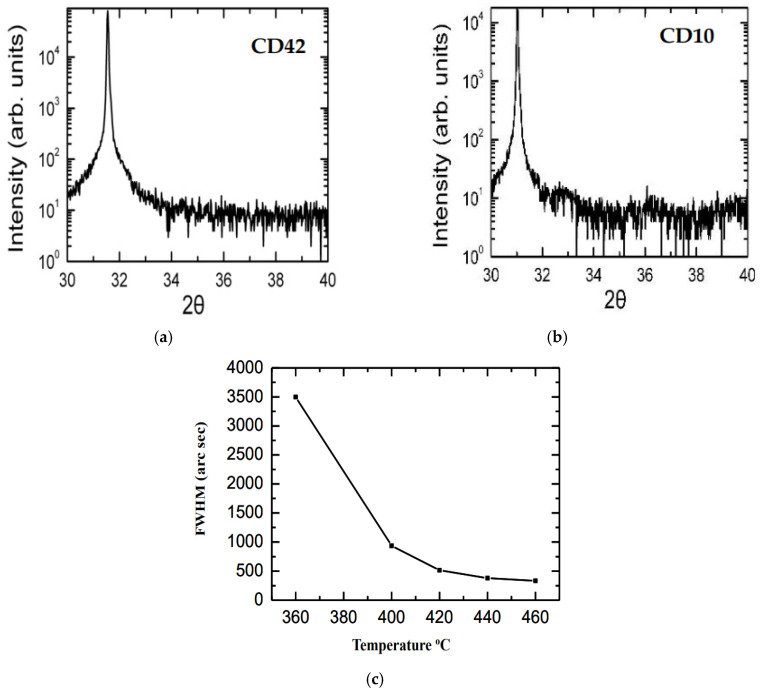
By using Philips x-pert four-axis HR-XRD equipment, we have reported the (0002) 2θ scan of two different PA-MBE-grown InN/Sapphire samples (**a**) CD42 and (**b**) CD10. (**c**) The results show the variation in full width at half maximum (FWHM) for the X-ray rocking curves with the growth temperature. It has clearly indicated that a higher growth temperature leads to better crystalline quality with a narrower XRC peak obtaining a well-ordered InN/Sapphire crystal structure.

The crystal structure of InN/Sapphire epifilms was obtained from the X-ray rocking curves (XRCs) by using a Philips x-pert four-axis HR-XRD system (Philips, Amsterdam, The Netherlands) [31]. In all samples, the expected diffraction peak corresponding to the wz InN (0002) plane at 2θ (~31.7°) was noticed to exhibit the XRC values of full width at half maximum (FWHM) in the range of 400 to 500 arc s. In Figure 6a,b, we have displayed our results of the HR-XRD measurements for two different CD42 and CD10 samples, respectively. The plot reported in Figure 6c has indicated the decrease in FWHM with an increase in the growth temperature. This result has certainly suggested that higher growth temperature leads to better crystalline quality with the narrower XRC peak used for achieving well-ordered InN/Sapphire epifilms.

### 4.2. Optical and Absorption Characteristics of InN/Sapphire

**Figure 7:**
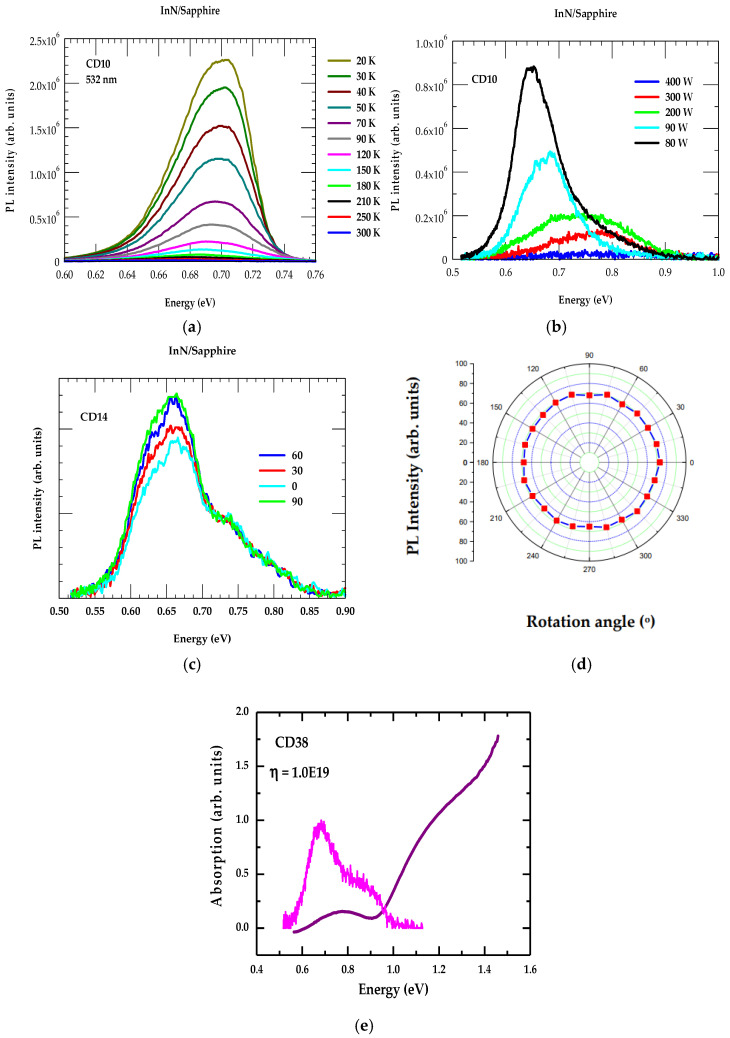
(**a**) Temperature-dependent PL spectra for sample CD10, (**b**) power-dependent PL spectra for sample CD10, (**c**) polarizer angle-dependent PL data for sample CD14 between 0° and 90°, (**d**) the polarization, and (**e**) PL (magenta color) and absorption (violet color line) spectra of InN/Sapphire sample CD38 measured at low temperature.

To appraise the electronic and vibrational properties of PA-MBE-grown samples, we have employed a Jobin Yvon TRIAX-320 system (Kyoto, Japan) equipped with a grating blazed at 2 μm. Measurements were performed by exciting a frequency-doubled Nd^+^ -YAG laser (λ = 532 nm) at room temperature (RT). A lead sulfide (PbS) detector was used for detection in the spectral range of 0.5–3 mm. Optical characteristics of InN/Sapphire films were acquired by performing PL measurements, in the temperature range of 20 K to 300 K. In Figure 7a,b, we have reported our temperature and power-dependent PL results, respectively, on one of the PA-MBE-grown CD10 samples. The PL data were collected between 20 K and 300 K on samples prepared at substrate temperature T (≡360 °C) and pressure (≡3 × 10^−5^ Torr) for 60 min keeping the plasma power at 200 W.

#### 4.2.1. Photoluminescence

Figure 7a reveals the intensity of the PL peak since it is narrow at lower T but widens, exhibiting a shoulder as T increases. Our measurements have perceived an estimated value of $E_{g}$ falling between ~0.67 eV to ~0.71 eV. The results are consistent with the observations made in recent years by several other groups [29,30,31,32].

In Figure 7a, one may also notice that hardly any peak is visibly shifted with T. The temperature stability of InN films and using them to design different optical device structures could be a big advantage. In Figure 7c,d, we have reported the RT PL spectra by exploiting a 532 nm excitation source on a CD14 sample with the polarizer angles varying between 0° and 90° and polarization, respectively. As the plasma power increases (see Figure 7b), more electrons become excited at the conduction band, leading to a “band filling” phenomenon where higher energy states in the conduction band become occupied, causing a shift in the PL peak towards higher energies [31]. Similar trends were observed in other samples. 

#### 4.2.2. Absorption

Figure 7a indicates that the asymmetric PL band shape is related to the existence of charge carrier concentration and Urbach tails [75,76]. The violet color line in Figure 7e is the absorption spectra measured at a low temperature. The magenta color inset is the room temperature PL spectra. The absorption edge is larger than the PL peak energy because the absorption at low T occurs significantly above the Fermi level. The simultaneous observation of the absorption edge and PL at essentially the same energy indicates that this energy position corresponds to the transition across the fundamental band gap of InN.

### 4.3. Raman Scattering

**Figure 8:**
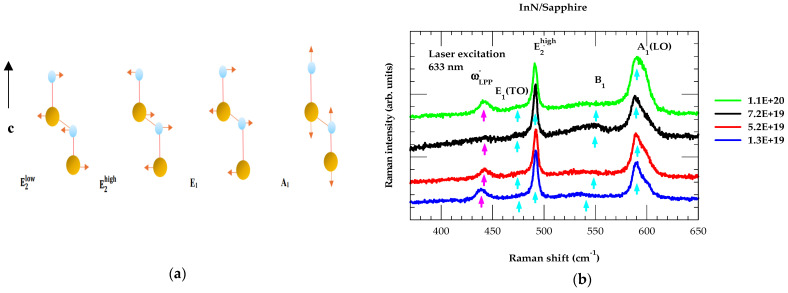
(**a**) Atomic displacements of Raman active modes in wurtzite InN. The large, orange-colored spheres represent In cations while the small sky blue-colored spheres represent the N anions. (**b**) Room temperature first-order Raman spectra obtained with 633 nm excitation source on four different InN/sapphire samples. Light, blue-colored arrows indicate InN phonon modes, while magenta-colored lines show low-frequency LO plasmon coupled phonon $\omega_{LPP}^{-}$.

Raman scattering spectroscopy is a highly valuable tool for investigating the vibrational characteristics of binary and/or ternary alloy semiconductors. Numerous studies on III-Ns are reported to identify the vibrational properties of hexagonal thin films [57]. For wz InN, the point group $C_{6v}$ symmetry exhibits six irreducible representations $A_{1}$, $A_{2},\;E_{1}^{2},\;E_{2}^{2},\;B_{1},$ and $B_{2}$, respectively, where the superscript indicates the mode degeneracy. While the $A_{1}$, $E_{1},$ and $E_{2}$ modes (see Figure 8a) are Raman active, the $A_{1}$ and $E_{1}$ phonons are also IR active [57]. To assess the different phonon features, a thermal micro-Raman system was employed with a He-Ne laser of wavelength 633 nm as an excitation source. In Figure 8b, the reported results of first-order Raman measurements were recorded in the backscattering configuration on four different PA-MBE-grown InN/Saphire samples with varied film thickness and charge carrier concentrations.

In hexagonal InN, one expects to observe three types of excitations: (a) the zone-center ($\vec{q}$ = 0) nonpolar ($E_{2}^{low}$ and $E_{2}^{high}$) modes, and transverse optical TO ($A_{1}$ and $E_{1}$) polar phonons, excited by deformation potential mechanism [57]; (b) the large wave vector ($\vec{q}$ ≠ 0) polar ($A_{1}$ and $E_{1}$) longitudinal optical LO modes caused by photon energies via a double-resonance excitation process [64]; and (c) the low-frequency long-wavelength ($\vec{q}$ ∼ 0) branch of LO phonon plasmon (LPP)-coupled $\omega_{LPP}^{-}$ mode. In highly disordered InN/Sapphire epifilms, we noticed the absence of a few Raman active phonons e.g., $E_{2}^{low}$, $A_{1}(TO)$ modes. We believe that disorder has triggered two major effects on Raman spectra: (i) initiating broadening of Raman active modes due to lattice distortions by widening the $B_{1}$, $E_{2}^{high}$, $A_{1}$(LO), and $\omega_{LPP}^{-}$ modes and (ii) affecting the spectral traits of certain optical phonons via interaction with charge carrier concentration. The Raman peaks observed near ~580 cm^−1^, ~490 cm^−1^ are attributed to $A_{1}(LO)$ and $E_{2}^{high}$ mode, respectively. These are in good agreement with published results from the literature [56,58]. In Table 2, we have reported our Raman scattering results (see Figure 8b) and compared/contrasted them with the existing experimental [60,61,62,63,64] and lattice dynamical [65,66,67] calculations.

It is to be noted that for an ideal wz InN crystal, $B_{1}$ is a silent mode. However, in disordered InN samples, it is observed as a weak feature occurring between the frequencies of $A_{1}(LO)$ and $E_{2}^{high}$ mode [57]. A peak revealed at a frequency below the forbidden A_1_(TO) mode in our measurements has clearly exhibited an upward frequency shift with the increase in η. This feature is unambiguously assigned as a $\omega_{LPP}^{-}$ mode. Phonons observed in Raman scattering [60,61,62,63,64] are confirmed by the lattice dynamical calculations [65,66,67]. Besides Raman experiments, we have also studied the phonon characteristics of InN/Sapphire samples by using far infrared reflectivity measurements.

### 4.4. Infrared Reflectivity

**Figure 9:**
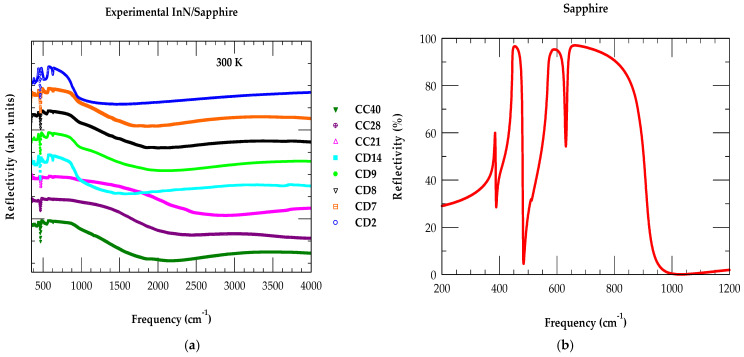
(**a**) The experimental FTIR reflectivity spectra recorded on eight different InN/Sapphire epilayer samples of various thicknesses d and charge carrier concentrations (see Table 1), (**b**) experimental measurement of the reflectivity spectra for hexagonal sapphire (Al_2_O_3_) substrate (see text).

The Bruker IFS 120 v/S FTIR spectrometer equipped with a specified sample stage was used to measure the reflectance and/or transmittance spectra. Here, we have considered a reflectance mode when collecting the results for all PA-MBE-grown InN/Sapphire epifilms. Measurements were performed at a near-normal incidence in the spectral range of 300 cm^−1^–4000 cm^−1^ with a 2 cm^−1^ resolution. A Globar source and deuterated triglycine sulfate (DTGS) detector were employed by combining a polyethylene window to record the s-polarized reflectance spectra on samples of different thicknesses and charge carrier concentrations (see Figure 9a).

The FIR reflectivity measurements on the InN/Sapphire epifilms were carefully performed by using a KRS-5 polarizer with the light incident on each sample at a fixed angle of 8°. To compensate for the atmospheric absorption, a reference spectrum was obtained from a gold-plated mirror mounted at the same surface height on the sample stage, prior to each experiment on the InN/Sapphire epifilms. Again, consistent with many other reports [62,63,64,65,66,67] available in the literature, our reflectivity measurement on Sapphire (see Figure 9b) has shown a complex phonon spectrum. The reflectivity spectrum of Sapphire is required to simulate the FTIR reflectivity spectra of InN/Sapphire epifilms using a “three-phase-model” (cf. Section 5.2).

Previously, Harima [57] used Raman scattering spectroscopy to measure the $\omega_{LPP}^{\pm }$ modes in doped bulk GaN materials, while Kasic et al. [64] exploited IR spectroscopic ellipsometry. Here, we have employed the FTIR and Raman spectroscopy to extract information on the $\omega_{LPP}^{-}$ modes in InN/Sapphire epifilms. An appropriate macroscopic model is adopted to simulate the dielectric functions in the far infrared region to calculate the reflectivity spectra (cf. Section 5.2). A comparison of theoretical results with experimental data has provided valuable information on phonon modes, film thickness d, surface interface roughness, η, and μ. Our results of $\omega_{LPP}^{-}$ modes in InN/Sapphire agreed reasonably well with Raman measurements [60,61,62,63,64] and other existing theoretical [65,66,67] and experimental data [57].

## 5. Theoretical Analysis of Optical and Phonon Data

Experimental measurements of the optical and phonon properties of the PA-MBE-grown InN/Sapphire samples were obtained earlier in Section 4 by using PL, RSS, and FTIR spectroscopies. In Section 5.1, Section 5.2 and Section 5.3, we have reported our systematic simulations to comprehend the experimental results.

### 5.1. Analysis of Optical Results: Varshni Model

**Figure 10:**
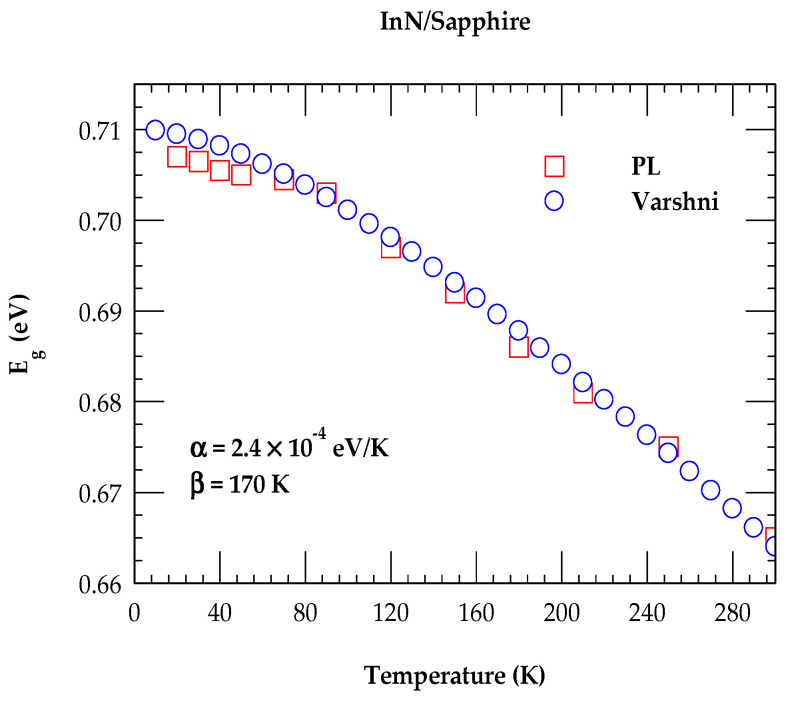
A comparison of the experimental (open, red-colored squares) T-dependent energy bandgap of the InN/Sapphire sample CD10 with Varshni’s (Ref. [77]) formula (blue open circles). An appropriate set of adjustable parameter values of α and β required in the fitting are also listed.

An empirical Varshni approach [77] has been widely adopted in semiconductors for describing the temperature dependence of PL (TD-PL) peak positions by using

(1) $$E_{g}\left(T\right)=E_{g}\left(T\to 0\right)-\frac{\alpha T^{2}}{T+\beta },$$

where $E_{g}\left(T\to 0\right)$ corresponds to the PL peak energy at T → 0; the terms α and β are the fitting parameters characteristic of a given material. With the choice of α = 2.4 × 10^−4^ eV/K and β = 210 K for the CD10 InN/Sapphire sample, Figure 10 shows a well-fitted result of our TD-PL experimental data describing a decrease in $E_{g}$ with an increase in T.

### 5.2. Analysis of Phonon Spectra in the FIR Region

A variety of models are available [68,69,70,71,72,73,74] with different levels of complexity for simulating the FTIR reflectivity spectra and comprehending the line shapes of the η-dependent $\omega_{LPP}^{\pm }$ modes. The simplest approach in semiconductors is based on the Drude susceptibility method [68]. The dielectric function $\tilde{\varepsilon }\left(\omega \right)$ provides a measure of how the dielectric medium responds to ω-dependent electric field in a homogeneous solid. It can be expressed with contributions of lattice vibrations ${\tilde{\varepsilon }}_{lat}\left(\omega \right)$ and plasmons ${\tilde{\varepsilon }}_{P}(\omega )$ [68]:

(2a) $$\tilde{\varepsilon }\left(\omega \right)=\varepsilon_{\infty }+\frac{(\varepsilon_{0}-\varepsilon_{\infty })\omega_{A1(TO)}^{2}}{\omega_{A1(TO)}^{2}-\omega^{2}-i\omega \gamma_{A1(TO)}}-\frac{\varepsilon_{\infty }\omega_{P}^{2}}{\omega^{2}+i\omega \gamma_{P}}={\tilde{\varepsilon }}_{lat}(\omega )+{\tilde{\varepsilon }}_{P}(\omega ),$$

(2b) $$\mathrm{with}\;{\tilde{\varepsilon }}_{lat}\left(\omega \right)=\varepsilon_{\infty }+\frac{S_{A1(TO)}\omega_{A1(TO)}^{2}}{\omega_{A1(TO)}^{2}-\omega^{2}-i\omega \gamma_{A1(TO)}}\;\mathrm{and}\;{\tilde{\varepsilon }}_{P}\left(\omega \right)=-\frac{\varepsilon_{\infty }\omega_{P}^{2}}{\omega^{2}+i\omega \gamma_{P}}.$$

The term ω in Equation (2a) is the frequency of incident light; $\varepsilon_{\infty }$ is a high-frequency dielectric constant; $\omega_{P}(\equiv \sqrt{\frac{4\pi \eta e^{2}}{{m_{e}}^{*}\varepsilon_{\infty }}})$ is the characteristic plasma frequency of conducting electron charge carriers which depends on its concentration η and effective mass ${m_{e}}^{*}$; $\gamma_{P}$($\gamma_{A1(TO)}$) signifies the plasma (phonon)-damping coefficient; $\omega_{A1(TO)}$ ($\omega_{A1(LO)}$) symbolizes the TO (LO) phonon frequency near the center of the Brillouin zone (BZ) (i.e., $\vec{q}$ → 0); and the mobility $\mu \left(\eta \right)$ of free-charge carriers is related to $\mu \left(\eta \right)(\equiv \frac{e}{{m_{e}}^{*}\gamma_{P}\left(\eta \right)})$.

Again, the dielectric function (cf. Equation (2a)) of InN material can be separated $\tilde{\varepsilon }\left(\omega \right)$ [≡$\varepsilon_{1}+i\varepsilon_{2}$] into its real [$\varepsilon_{1}$(ω)] and imaginary [$\varepsilon_{2}$(ω)] parts [68,69,70,71,72,73,74]. The term $\varepsilon_{2}$(ω) represents absorption as a function of ω and affects the reflectivity (transmission) spectrum of the material. Again, $\tilde{\varepsilon }\left(\omega \right)$ can be related to the real and imaginary parts of the complex refractive index using $\tilde{n}$ [≡n + iκ] = $\sqrt{\tilde{\varepsilon }\left(\omega \right)}$

(3) $$n={\left[\frac{{\left(\varepsilon_{1}^{2}+\varepsilon_{2}^{2}\right)}^{1/2}+\varepsilon_{1}}{2}\right]}^{1/2},\;\kappa ={\left[\frac{{\left(\varepsilon_{1}^{2}+\varepsilon_{2}^{2}\right)}^{1/2}-\varepsilon_{1}}{2}\right]}^{1/2}=\left(\frac{\varepsilon_{2}}{2n}\right),$$

where n, κ in Equation (3) are, respectively, the index of refraction and extinction coefficients. 

The reflectivity R(ω) spectra of bulk InN material in the FIR region can be expressed [68,69,70,71,72,73,74] in terms of its reflectance coefficient $\tilde{r}\left(\omega \right)$

(4) $$R\left(\omega \right)={\left|\tilde{r}\left(\omega \right)\right|}^{2}={\left[\frac{\sqrt{\tilde{\varepsilon }\left(\omega \right)}-1}{\sqrt{\tilde{\varepsilon }\left(\omega \right)}+1}\right]}^{2}=\left[\frac{{\left(n-1\right)}^{2}+\kappa^{2}}{{\left(n+1\right)}^{2}+\kappa^{2}}\right],$$

In InN/Sapphire epifilms, the simulation of IR reflectance is one of the bases of multiple reflections in epilayers for causing the interferences between the reflected (transmitted) radiations. Theoretical calculations are performed in a “three-phase-model” using the dielectric functions for air ${\tilde{\varepsilon }}_{1}=1$ (air), 2 ${\tilde{\varepsilon }}_{2}={\tilde{\varepsilon }}_{tf}$ (InN thin-film), and 3 ${\tilde{\varepsilon }}_{3}={\tilde{\varepsilon }}_{s}$ (Sapphire substrate). One must note that the s-polarized spectra combine only with the component of dielectric function parallel to the plane of layers, while the p-polarization spectra couple simultaneously to the components parallel and perpendicular to the plane of layers. Relative contributions of s- and p-components can be determined from the angle of incidence θ_i_. In near-normal conditions (θ_i_ ≈ 0), the total reflectance $R\left(\omega \right)$ is expressed as a mean value of the s- and p-polarized reflection coefficients [68,69,70,71,72,73,74]:

(5) $$R\left(\omega \right)=\frac{\left|{\tilde{r}}_{123s}^{2}+{\tilde{r}}_{123p}^{2}\right|}{2},$$

where ${\tilde{r}}_{123a}$ with a (≡s- and p-) polarization; the numbers 1, 2, and 3 signify the air, film, and substrate, respectively [68,69,70,71,72,73,74]. For an epifilm of thickness d and following Cadman and Sadowski [74], one can evaluate ${\tilde{r}}_{123a}$ by using the following equation:

(6) $${\tilde{r}}_{123a}=\frac{{\tilde{r}}_{12a}+{\tilde{r}}_{23a}\exp[i2\beta ]}{1+{\tilde{r}}_{12a}{\tilde{r}}_{23a}\exp[i2\beta ]},$$

in terms of the Fresnel coefficients ${\tilde{r}}_{ija}=\frac{{\tilde{n}}_{ia}-{\tilde{n}}_{ja}}{{\tilde{n}}_{ia}+{\tilde{n}}_{ja}}$ and phase multiplier $\beta =2\pi d\omega \sqrt{{\tilde{\varepsilon }}_{2}}$. The above approach has been used for simulating the IR reflectivity spectra at a near-normal incidence (θ_i_ ≈ 0) in the heteroepitaxial films.

**Figure 11:**
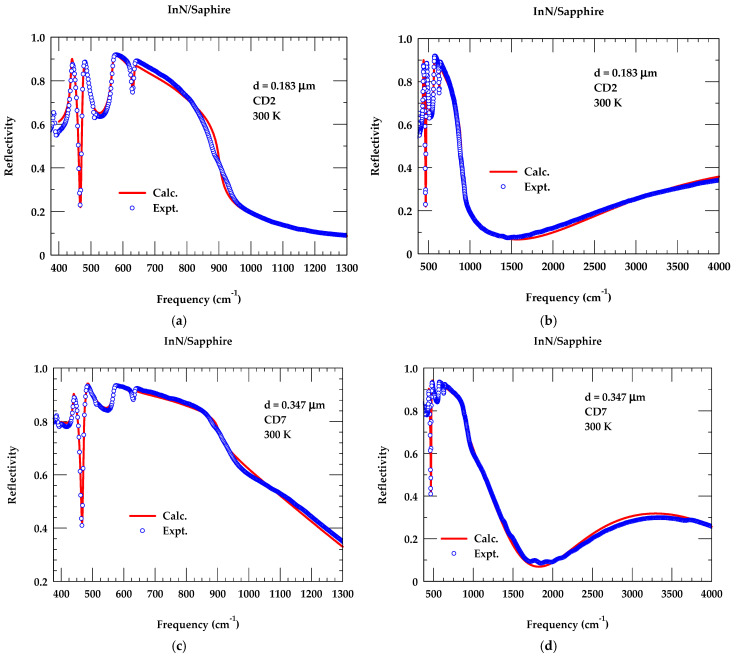
A comparison of the experimental (open squares) room temperature infrared reflectivity spectra of PA-MBE-grown InN/sapphire samples with the theoretical results (full lines) using the parameter values of Table 3 (**a**) for sample CD2 between 350 and 1300 cm^−1^, and (**b**) between 350 and 4000 cm^−1^. (**c**,**d**) same as (**a**,**b**) but for sample CD7.

By using the set of parameter values reported in Table 3, and adopting the methodology outlined above, we have systematically simulated the reflectivity spectra of all the PA-MBE-grown InN/Sapphire samples of different thickness d, and charge carrier concentration, η. The calculated FTIR reflectivity spectra are compared with the experimental data in Figure 11a–d for CD2 and CD7 samples of different d and η. The perusal of results in different frequency regions for sample CD2 (see Figure 11a,b) provided a very good agreement with the experimental data, confirming the quality of our PA-MBE-grown InN/Sapphire epilayers. The values of η and μ are estimated from the FTIR-fitting procedures. The results are compared/contrasted in Table 1 with η, μ data obtained by Hall measurements. Similar results are reported in Figure 11c,d for the CD7 sample.

### 5.3. LO Phonon Plasmon Coupled Modes

Neglecting the phonon $\gamma_{A1(TO)}$ and plasmon $\gamma_{P}$ damping factors, the zeroes of the real part of the dielectric function (Equation (2b)) yield the frequencies of $\omega_{LPP}^{\pm }$ modes [68]:

(7) $$\omega_{LPP}^{\pm }={\left\{\frac{1}{2}\left[\omega_{A1(LO)}^{2}+\omega_{P}^{2}\pm \sqrt{(\omega_{A1(LO)}^{2}+\omega_{P}^{2}{)}^{2}-4\omega_{P}^{2}\omega_{A1(TO)}^{2}}\right]\right\}}^{1/2},$$

where $\omega_{LPP}^{+}$> $\omega_{A1(LO)}$ and $\omega_{LPP}^{-}$< $\omega_{A1(TO)}$.

**Figure 12:**
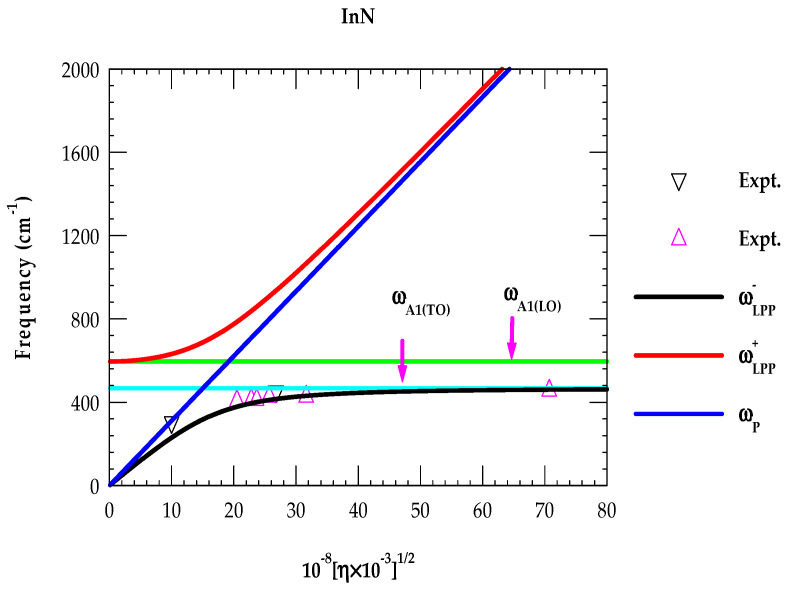
Calculated phonon frequencies as a function of the charge carrier concentration in InN. The sky blue-colored line represents $\omega_{A1(TO)}$ phonon while the green-colored line signifies the $\omega_{A1\left(LO\right)}$ mode frequency. The blue-colored line represents the plasma frequency; the black-colored line indicates low-frequency LO phonon–plasmon coupled modes $\omega_{LPP}^{-}$, while the red colored line indicates high-frequency $\omega_{LPP}^{+}$ modes, respectively. Low-frequency $\omega_{LPP}^{-}$ modes observed in Raman scattering measurements (shown by a black-colored inverted triangle and magenta-colored triangles) as a function of a free-charge carrier compared reasonably well with our theoretical results (see text).

In doped polar materials, the coupling of free-charge carriers via their macroscopic electric fields with LO phonons give rise to the $\omega_{LPP}^{\pm }$ modes. For n-doped InN, we have displayed our calculated results in Figure 12 as a function of η.

The $\omega_{LPP}^{-}$ phonon frequencies (black-colored inverted triangle and magenta-colored triangles) were estimated from our Raman scattering measurements on n-InN epilayers as well as reported by others [57]. In heavily doped n-type InN samples with high mobility, the $\omega_{LPP}^{+}$ branch exhibits a plasmon like character while $\omega_{LPP}^{-}$ branch displays a phonon-like feature. The frequency of the later type of mode (i.e., $\omega_{LPP}^{-}$) approaches to the $\omega_{A1(TO)}$ phonon value (see Figure 12).

Several mechanisms are expected to contribute to light scattering by $\omega_{LPP}^{\pm }$ modes [57]. These include the following: (a) the modulation of crystal potential by atomic displacements and (b) the Fröhlich interaction involving macroscopic electric fields. Here, in the n-type InN we have focused only on the observed charge carrier density η dependence of $\omega_{LPP}^{-}$ as $\omega_{LPP}^{+}$ modes are missing in our Raman scattering measurements (cf. Figure 8b). The absence of $\omega_{LPP}^{+}$ can be elucidated either to the inhomogeneous broadening caused by fluctuations of the charge-carrier density η in the PA-MBE-grown samples and/or to the low signal levels. Such characteristics in Raman scattering spectroscopy predominantly affect the $\omega_{LPP}^{+}$ modes as compared to the phonon-like $\omega_{LPP}^{-}$ modes. In Figure 12, we have included $\omega_{LPP}^{-}$ mode frequencies (a black-colored inverted triangle and magenta-colored triangles) estimated in our Raman scattering measurements on n-InN epilayers and revealed by others [57].

## 6. Concluding Remarks

In conclusion, we have reported the results of our systematic experimental measurements and theoretical simulations to extract the structural, optical, and phonon properties on a series of narrow bandgap InN/Sapphire epifilms grown by the PA-MBE technique. Most of our samples exhibited higher charge carrier concentrations due to the inherent nature of the growth process which could have introduced native defects to act as electron donors. The Hall effect measurements have confirmed it. Again, the preparation of InN/Sapphire films by epitaxial methods is quite a delicate process. It requires carefully monitoring the growth temperature T, plasma power, N/In ratio, and pressure, P. One must note that the crystal qualities of epifilms, including the polarity, are sensitive to the nitridation procedures. Here, we have systematically studied the impact of N-plasma power on InN growth at T above its decomposition temperature. Due to the reduced distance between the N atoms at higher P, one expects N-flow kinetics, diffusion, surface components, and scattering rates to change in the growth chamber which could impact on the quality of InN films. Based on our methodical studies we believe that ionized N, rather than the molecular or neutral species, is responsible for controlling the InN/Sapphire growth and optimizing the surface morphology. Temperature- and power-dependent photoluminescence measurements are also performed to assess the bandgap variation (~0.60–0.80 eV) in all the PA-MBE-grown samples. Our high-resolution X-ray diffraction studies have clearly indicated that the increase in growth temperature resulted in the observed narrow X-ray-rocking curve peaks leading to better-quality films with well-ordered crystalline structures. Careful simulations of the Fourier transform infrared reflectivity spectra have provided the values of η and μ in good agreement with the Hall measurements. The complementary measurements using first-order Raman scattering spectra have not only identified the accurate values of phonon features in InN/Sapphire samples but also revealed the low-frequency longitudinal optical phonon plasmon coupled mode $\omega_{LPP}^{-}$ in excellent agreement with our theoretical calculations.

## Figures and Tables

**Table 1:** PA-MBE growth conditions of the selected set of InN/Sapphire epifilm samples (see text).

					IR ^(a)^		Hall	
Samples Name	Substrate Temp. (°C)	In Temp. (°C)	Growth Pressure (Torr)	Thickness d (nm)	Carrier Conc. (×10^19^ cm^−3^)	Mobility (cm^2^/Vs)	Carrier Conc. (×10^19^ cm^−3^)	Mobility (cm^2^/Vs)
CD2	360	840	7 × 10^−5^	182.65	1.80	363.02	2.08	961.0
CD7	360	846	3 × 10^−5^	347.12	2.30	556.92	2.49	751.4
CD8	340	836	5 × 10^−5^	366.93	2.45	550.34	3.29	809.3
CD9	360	846	3 × 10^−5^	232.57	3.09	491.39	-	-
CD10	360	840	3 × 10^−5^	195.31	1.16	427.33	-	-
CD14	360	840	3 × 10^−5^	362.37	1.58	644.93	1.74	692.2
CD15	360	846	3 × 10^−5^	355.08	2.24	443.59	-	-
CD16	360	825	3 × 10^−5^	728.02	4.98	877.78	-	-
CC21	360	825	5 × 10^−5^	272.92	5.68	550.24	9.49	451.2
CC28	440	840	5 × 10^−5^	514.17	3.36	620.89	5.13	464.0
CC30	360	840	5 × 10^−5^	418.80	1.91	942.11	-	-
CC40	360	836	5 × 10^−5^	351.01	2.77	852.52	3.32	788.8

^(a)^ Estimated by fitting the IR spectra (cf. Section 5.2).

**Table 2:** Comparison of Raman modes (in cm^−1^) in PA-MBE InN/Sapphire films with other experimental and lattice dynamical calculations.

	Sample #1 ^(a)^	Sample #2 ^(a)^	Sample #3 ^(a)^	Sample #4 ^(a)^	Experimental ^(b)^	Lattice Dynamical ^(c)^
Mode	η = 1.3 × 10^19^	η = 5.2 × 10^19^	η = 7.2 × 10^19^	η = 1.1 × 10^20^		
$A_{1}$(LO)	590	590	590	590	580, 586, 588, 590, 596	586, 587, 589
$B_{1}$	540	540	550	550	540, 565	530, 566, 568, 570
$E_{2}^{\mathrm{high}}$	491	491	491	491	488, 490, 491, 495	483, 485, 492
$E_{1}$(TO)	475	477	477	477	472, 475, 476, 477	457,467, 470, 472
$\omega_{LPP}^{-}$	440	445	447	447		

^(a)^ Our; ^(b)^ Refs. [60,61,62,63,64]; ^(c)^ Refs. [65,66,67].

**Table 3:** Optical constants are used in Equations (2)–(5) to simulate the FTIR spectra of InN.

Samples	$S_{A1(TO)}$ (cm^−1^)	$\omega_{A1(TO)}$ (cm^−1^)	$\gamma_{A1(TO)}$ (cm^−1^)	$\omega_{P}$ (cm^−1^)	$\gamma_{P}$ (cm^−1^)
CD2	4.72	478.35	4.48	1385.37	256.67
CD7	4.91	478.70	4.53	1564.27	167.31
CD8	3.68	477.17	5.34	1615.79	169.30
CD9	6.18	479.21	5.15	1814.30	189.62
CD10	4.63	474.77	5.55	1108.99	218.04
CD14	2.65	473.91	6.36	1296.51	144.47
CD15	3.78	477.68	3.59	1543.39	210.05
CD16	5.63	475.68	5.08	2302.70	106.15
CC21	5.47	478.01	5.84	2457.15	169.34
CC28	4.87	477.65	5.56	1890.96	150.07
CC30	3.34	475.54	7.15	1425.87	98.90
CC40	5.11	478.33	5.09	1715.75	109.29

## Data Availability

The data that support the findings of this study are available from the corresponding author upon reasonable request.
